# Radiological review of prior screening mammograms of screen-detected breast cancer

**DOI:** 10.1007/s00330-020-07130-y

**Published:** 2020-10-01

**Authors:** Tone Hovda, Kaitlyn Tsuruda, Solveig Roth Hoff, Kristine Kleivi Sahlberg, Solveig Hofvind

**Affiliations:** 1 Department of Radiology, Vestre Viken Hospital Trust, PO Box 800, 3004 Drammen, Norway; 2 Institute of Clinical Medicine, University of Oslo, PO Box 1171, Blindern, 0318 Oslo, Norway; 3 Section for Breast Cancer Screening, Cancer Registry of Norway, PO Box 5313, Majorstuen, 0304 Oslo, Norway; 4 Oslo Centre for Biostatistics and Epidemiology, Department of Biostatistics, Institute of Basic Medical Sciences, University of Oslo, PO Box 1122, Blindern, 0317 Oslo, Norway; 5 Department of Radiology, Ålesund Hospital, Møre og Romsdal Hospital Trust, Åsehaugen 5, 6017 Ålesund, Norway; 6 Department of Circulation and Medical Imaging, Faculty of Medicine and Health Sciences, NTNU, Trondheim, Norway; 7 Department of Research and Innovation, Vestre Viken Hospital Trust, PO Box 800, 3004 Drammen, Norway; 8 Department of Cancer Genetics, Institute for Cancer Research, Oslo University Hospital Trust, PO Box 4950, 0424 Oslo, Norway; 9 Faculty of Health Science, Oslo Metropolitan University, PO Box 4, St. Olavs Plass, 0130 Oslo, Norway

**Keywords:** Mass screening, Breast neoplasm, Digital mammography, Mammography, Female

## Abstract

**Objective:**

To perform a radiological review of mammograms from prior screening and diagnosis of screen-detected breast cancer in BreastScreen Norway, a population-based screening program.

**Methods:**

We performed a consensus-based informed review of mammograms from prior screening and diagnosis for screen-detected breast cancers. Mammographic density and findings on screening and diagnostic mammograms were classified according to the Breast Imaging-Reporting and Data System®. Cases were classified based on visible findings on prior screening mammograms as true (no findings), missed (obvious findings), minimal signs (minor/non-specific findings), or occult (no findings at diagnosis). Histopathologic tumor characteristics were extracted from the Cancer Registry of Norway. The Bonferroni correction was used to adjust for multiple testing; *p* < 0.001 was considered statistically significant.

**Results:**

The study included mammograms for 1225 women with screen-detected breast cancer. Mean age was 62 years ± 5 (SD); 46% (567/1225) were classified as true, 22% (266/1225) as missed, and 32% (392/1225) as minimal signs. No difference in mammographic density was observed between the classification categories. At diagnosis, 59% (336/567) of true and 70% (185/266) of missed cancers were classified as masses (*p* = 0.004). The percentage of histological grade 3 cancers was higher for true (30% (138/469)) than for missed (14% (33/234)) cancers (*p* < 0.001). Estrogen receptor positivity was observed in 86% (387/469) of true and 95% (215/234) of missed (*p* < 0.001) cancers.

**Conclusions:**

We classified 22% of the screen-detected cancers as missed based on a review of prior screening mammograms with diagnostic images available. One main goal of the study was quality improvement of radiologists’ performance and the program. Visible findings on prior screening mammograms were not necessarily indicative of screening failure.

**Key Points:**

• *After a consensus-based informed review, 46% of screen-detected breast cancers were classified as true, 22% as missed, and 32% as minimal signs.*

• *Less favorable prognostic and predictive tumor characteristics were observed in true screen-detected breast cancer compared with missed.*

• *The most frequent mammographic finding for all classification categories at the time of diagnosis was mass, while the most frequent mammographic finding on prior screening mammograms was a mass for missed cancers and asymmetry for minimal signs.*

## Introduction

Breast cancer is diagnosed among screening participants as screen-detected breast cancer or interval breast cancer (breast cancer diagnosed between two scheduled screening rounds after a negative screening episode). When obvious mammographic findings corresponding to the location of the tumor are visible on prior screening mammograms, which in retrospect should have resulted in a recall, the cancer may be defined as missed. In studies, up to 50% of interval and screen-detected breast cancers can present visible findings on prior screening mammograms, ranging from minor benign-looking findings to obviously missed cancers [1–8]. Breast cancer can be missed at screening due to misperception—the lesion is not perceived by the radiologist—or misinterpretation—the lesion is detected by the radiologist, but not considered suspicious enough to warrant a recall, either by the reading radiologist or at a consensus meeting. Further, unsatisfactory image quality, positioning, or inadequate assessment at recall may cause a cancer to be missed [9, 10]. Mammographic density may also impact the rates of missed breast cancer due to the masking effect of dense breast tissue and overlapping structures [11].

Radiologic reviews may be useful for quality assurance and quality improvement of both the program and the radiologists. European guidelines [12] and BreastScreen Norway quality manual [13] recommend continuous surveillance and regular review of screening mammograms performed prior to diagnosis of interval breast cancer. Further, in the National Health Service Breast Screening Programme (UK), radiologists are obliged to audit mammograms of interval breast cancer [14, 15]. Use of audits is discussed also in other countries, and we expect this topic to receive more attention in the future [16]. However, the information available as well as the number of reviewers affect the results of the review [3, 4]. Further, whether a commitment to inform the women will affect the results of a review or an audit is debatable.

Several review studies of interval breast cancer have been performed [8, 17–20]. Larger review studies of screen-detected breast cancer, particularly including digital mammography (DM), are, to our knowledge, sparse. Screen-detected breast cancer with no visible findings on prior screening mammograms, defined as true cases, may grow faster than missed breast cancer. Thus, different histopathological characteristics and different mammographic findings are anticipated for true versus missed screen-detected breast cancers.

We conducted a nationwide consensus-based, informed review within BreastScreen Norway. The study included prior screening mammograms and mammograms available at diagnosis from women diagnosed with screen-detected breast cancer. The overall aim of the study was quality improvement for radiologists’ performance and the program as such. The objectives were to investigate the proportions of true and missed screen-detected breast cancers and to explore whether mammographic findings, density, or histopathological characteristics differed between the two groups. We hypothesized that these three aspects differed between true and missed screen-detected breast cancers.

## Materials and methods

The study was approved by the data protection official at the Cancer Registry of Norway (CRN) (PVO approval number: 2016/4696), and the local breast centers agreed to the study. The Cancer Registry Regulation waived the requirement to obtain written informed consent for use of screening data for quality assurance and research [21]. We received de-identified data for analyses from the CRN.

BreastScreen Norway offers women aged 50–69 biennial screening with two-view standard DM. The screening exams take place at 27 stationary or mobile units. Screen reading is performed at 16 breast centers and includes independent double reading by breast radiologists. The radiologists score each breast on a 5-point scale; 1 indicates negative findings, and 5 indicates a high suspicion for malignancy. Exams scored ≥ 2 by either radiologist are discussed in a consensus meeting to decide whether to recall the woman [22]. The median annual reading volume for radiologists during 1996–2016 was 4492 exams; 46% of the radiologists had over 10 years of screen reading experience [23].

### Materials and review logistics

CRN randomly extracted 85 screen-detected breast cancer cases from each of the 16 breast centers. All examinations were performed with DM during 2006–2015, and all women had a screening exam with DM 2 years previously (prior screen). We aimed to review at least 75 cases at each center within an 8-h session, including instruction time. Panels of 5 breast radiologists performed the review from September 2016 to April 2017. Radiologists not participating in the panel could observe the review session.

We performed a consensus-based, fully informed retrospective radiological review. All screening and diagnostic images were available to the reviewers, including ultrasound and MRI, as well as histopathological reports. The breast centers were divided into 8 pairs; two radiologists from each center reviewed each other’s images and made up the consensus panel together with one independent radiologist, the first author (T.H.). To qualify for the panel, we required the radiologists to have at least 1 year of experience in screen reading and a reading volume of ≥ 5000 mammograms during the past 2 years.

To ensure consistency across the centers in the review procedures, classifications, registration, and coding of results, T.H. took part in all reviews together with a representative from CRN. Ahead of each session, T.H. presented the classification systems and general instructions for the review. In the case of dissent among the panel members, a majority decision was made. All images were reviewed locally from the picture archiving and communication system (PACS).

### Review procedure

**Fig. 1:**
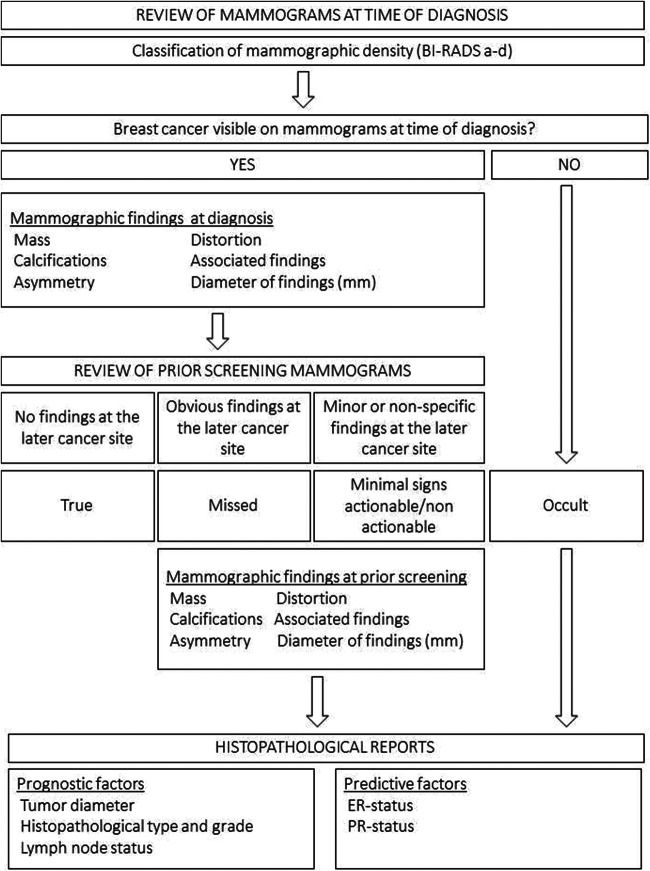
Review procedure

The review procedure is described in Fig. 1. First, we reviewed the mammograms, resulting in recall and diagnosis of screen-detected breast cancer, and thereafter classified mammographic density using the Breast Imaging-Reporting and Data System (BI-RADS) 5th edition categories a–d [24]. We identified the malignancy and classified mammographic findings as mass, calcifications, asymmetry, distortion, or associated findings using the BI-RADS lexicon. If calcifications were present alongside another finding, the non-calcification finding was preferred for classification, unless calcifications were the dominant finding. We classified the largest tumor in case of multifocality or bilateral disease. We measured the diameter (mm) of the findings on the mammogram, using electronic calipers. If no malignancy was visible at the time of diagnosis, the case was classified as occult.

**Fig. 2:**
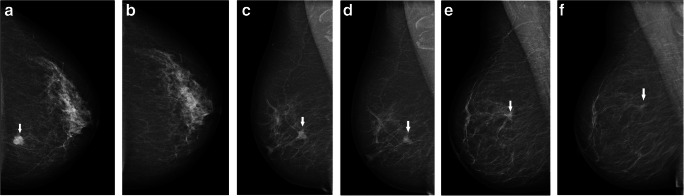
**a**, **b** True screen-detected breast cancer. A 57-year-old woman presenting with an irregular mass in the medial aspect of the left breast at screening (arrow), diagnosed with a 17-mm invasive carcinoma of no special type. Left craniocaudal view at diagnosis (**a**) and at prior screening (**b**). **c**, **d** Missed screen-detected breast cancer. A 67-year-old woman presenting with a spiculated mass in the upper lateral part of the right breast at screening (arrow), diagnosed with an 18-mm invasive lobular carcinoma. Right mediolateral oblique (MLO) view at diagnosis (**c**). A spiculated mass at the later cancer site (arrow) also appears at prior screening (**d**). **e**, **f** Minimal sign screen-detected breast cancer. A 67-year-old woman presenting with a spiculated mass in the upper outer quadrant at screening (arrow), right MLO view (**e**), diagnosed with a 17-mm invasive lobular carcinoma. A non-specific focal asymmetry (arrow) is visible on prior screening mammograms (**f**)

Thereafter, we reviewed and classified the finding, based on its visibility on prior screening mammograms. True cancers showed no findings at the eventual cancer site on prior screening mammograms (Fig. 2a, b). Cancers with obvious findings at the cancer site on priors which retrospectively should have resulted in a recall, as considered by the reviewing radiologists, were defined as missed (Fig. 2c, d). Minimal sign cancers showed minor findings on prior mammograms, not necessarily warranting assessment (Fig. 2e, f). At review, minimal signs were classified as either actionable (recall considered possible, but not expected within a screening program) or non-actionable (non-specific findings, recall not considered possible). However, we consider all minimal signs as one category in the main analyses. Finally, we classified mammographic findings on prior mammograms for missed and minimal sign cancers.

Histopathological information extracted from the CRN database was merged with data from the review and communicated to the radiologists after complete classification of each case. Prognostic characteristics included histological type (ductal carcinoma in situ (DCIS), invasive carcinoma of no special type (NST), invasive lobular carcinoma (ILC), other invasive carcinomas) and, for invasive cancers, also histological grade, histopathological tumor diameter (mm), and lymph node status. Predictive tumor characteristics for invasive cancers included estrogen receptor (ER) and progesterone receptor (PR) status.

### Statistical analyses

We performed descriptive analyses of age at diagnosis, review classification categories, mammographic findings, mammographic density, and histopathological characteristics. Data were presented as percentages with 95% confidence intervals (CIs), calculated using the Clopper-Pearson mid-P interval; means ± standard deviations (SDs); and medians with interquartile ranges (IQRs). Chi-square tests and independent sample *t* tests were used to test the differences between review classification categories and mammographic findings or histopathological characteristics, as well as between mammographic findings and histopathological characteristics. The Bonferroni correction was used to adjust for multiple testing, and a *p* value < 0.001 was considered statistically significant. IBM SPSS Statistics (version 25) was used for all analyses.

## Results

We reviewed and classified mammograms from 1227 women screened with DM, recalled due to mammographic findings and diagnosed with breast cancer. We excluded two mammographically occult cases. The final study sample thus consisted of 1225 cases.

Mean age at diagnosis was 62 ± 5 years, and median age was 63 years (59, 66). We classified 46% (567/1225) of the screen-detected cancers as true, 22% (266/1225) as missed, and 32% (392/1225) as minimal signs.

At the time of diagnosis, 59% (336/567) of true, 70% (185/266) of missed, and 65% (256/392) of minimal signs were masses (*p* = 0.004 for true versus missed), while 23% (128/567) of true, 17% (46/266) of missed, and 17% (68/392) of minimal signs were calcifications. The mean diameter of mammographic findings at diagnosis was 18 mm ± 14 for true, 20 mm ± 15 for missed, and 17 mm ± 16 for minimal signs (Table 1).

**Table 1:** Mammographic findings at diagnosis and on prior screening mammograms stratified by review classification categories

	Total		True		Missed		Minimal signs	
	*n* = 1225	100%	*n* = 567	46%	*n* = 266	22%	*n* = 392	32%
Age (years)								
Mean ± SD		62 years ± 5		62 years ± 5		63 years ± 5		62 years ± 5
Median (IQR)		63 years (59, 66)		63 years (58, 66)		64 years (60, 67)		63 years (59, 66)
Mammographic density								
BI-RADS a + b	810	66% (63, 69)	372	66% (62, 70)	178	67% (61, 73)	260	66% (61, 71)
BI-RADS c + d	415	34% (31, 37)	195	34% (31, 39)	88	33% (28, 39)	132	34% (29, 39)
Mammographic findings at diagnosis								
Mass	777	63% (61, 66)	336	59% (55, 63)	185	70% (66, 75)	256	65% (60, 70)
Calcifications	242	20% (18, 22)	128	23% (19, 26)	46	17% (13, 22)	68	17% (14, 22)
Asymmetry	106	9% (7, 10)	58	10% (8, 13)	15	6% (3, 9)	33	8% (6, 12)
Distortion	97	8% (7, 10)	43	8% (6, 10)	20	8% (5, 11)	34	9% (6, 12)
Associated findings	3	0.2% (0, 1)	2	0.4% (0, 1)			1	0.3% (0, 1)
Mammographic diameter								
Mean ± SD	18 mm ± 15		18 mm ± 14		20 mm ± 15		17 mm ± 16	
Median (IQR)	14 mm (10, 21)		14 mm (10, 20)		16 mm (11, 23)		14 mm (10, 21)	
Data not available	83		46		5		30	
Mammographic findings prior mammograms								
Mass	134	20% (17, 24)			115	43% (37, 49)	19	5% (3, 8)*
Calcifications	142	22% (19, 25)			62	23% (18, 29)	80	20% (17, 25)
Asymmetry	302	46% (42, 50)			66	25% (20, 31)	236	60% (55, 65)*
Distortion	78	12% (10, 15)			23	9% (6, 13)	55	14% (11, 18)
Associated findings	2	0.3% (0, 1)					2	0.5% (0, 2)
Mammographic diameter								
Mean ± SD	12 mm ± 11				14 mm ± 12		10 mm ± 9*	
Median (IQR)	9 mm (6, 14)				11 mm (7, 15)		8 mm (5, 12)	
Data not available		191				20		171

Unless otherwise specified, data are the number of patients and 95% confidence intervals in parenthesis

*SD* standard deviation, *IQR* interquartile range

*p < 0.001, compared with missed

At prior screening, 43% (115/266) of missed cancers and 5% (19/392) of minimal signs were masses (*p* < 0.001); 25% (66/266) of missed and 60% (236/392) of minimal signs presented as asymmetries (*p* < 0.001). The mean diameter of mammographic findings on prior screening mammograms was 14 mm ± 12 for missed and 10 mm ± 9 for minimal signs (*p* < 0.001) (Table 1).

**Fig. 3:**
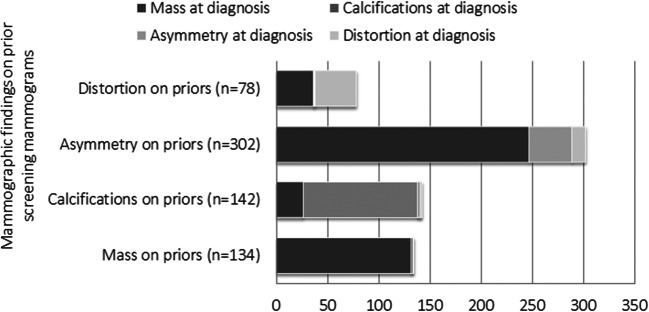
Mammographic findings on prior screening mammograms of missed and minimal sign cancers, stratified by mammographic findings on mammograms at diagnosis of screen-detected breast cancer

Eighty-two percent (247/302) of cases classified as asymmetries on prior mammograms were classified as masses at diagnosis. Ninety-nine percent (132/134) of the cases classified as masses at priors were classified as masses at diagnosis; 79% (112/142) of calcifications on priors also presented as such at diagnosis, and 18% (26/142) of calcifications presented as a mass. Among cancers classified as distortions on priors, 51% (40/78) were classified as distortions at diagnosis and 46% (36/78) as a mass (Fig. 3).

**Fig. 4:**
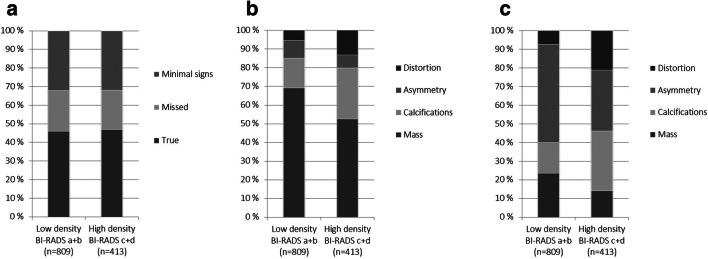
**a** Distribution of review classification groups based on findings on prior screening mammograms (true, missed, or minimal signs) stratified by the BI-RADS density score (low: BI-RADS a + b; high: BI-RADS c + d) (*p* = 0.88). **b** Distribution of mammographic findings at diagnosis (mass, calcifications, asymmetry, or distortion) stratified by the BI-RADS density score (*p* < 0.001). **c** Distribution of mammographic findings on prior screening mammograms stratified by the BI-RADS density score *p* < 0.001

We did not observe any differences in mammographic density between classification groups; the percentage of BI-RADS a + b was 66% (372/567) for true, 67% (178/266) for missed, and 66% (260/392) for minimal signs (Table 1, Fig. 4a). However, the percentages of calcifications and distortions were statistically higher in mammograms classified with high (BI-RADS c + d) mammographic density compared with low (BI-RADS a + b), both at diagnosis (Fig. 4b) and at prior screening (Fig. 4c).

No statistically significant differences were observed in the distribution of histopathological type for missed, true, or minimal signs; DCIS accounted for 17% (98/567) of the true, 12% (32/266) of the missed, and 13% (50/392) of the minimal signs (Table 2).

**Table 2:** Histopathological tumor characteristics stratified by review classification categories

	Total		True		Missed		Minimal signs	
	*n* = 1225	100%	*n* = 567		*n* = 266		*n* = 392	
Histopathological type								
DCIS	180	15% (13, 17)	98	17% (14, 21)	32	12% (8, 17)	50	13% (10, 17)
Invasive carcinoma of NST	880	72% (69, 74)	407	72% (68, 76)	194	73% (67, 78)	279	71% (66, 76)
ILC	94	8% (6, 9)	35	6% (4, 9)	21	8% (5, 12)	38	10% (7, 13)
Other invasive carcinomas	71	6% (5, 7)	27	5% (3, 7)	19	7% (4, 11)	25	6% (4, 9)
DCIS	180		98		32		50	
Tumor diameter								
Mean ± SD	20 mm ± 16		20 mm ± 14		18 mm ± 14		23 mm ± 21	
Median (IQR)	15 mm (10, 27)		15 mm (10, 26)		15 mm (8, 25)		17 mm (10, 33)	
Data not available	23		15		2		6	
Histological grade								
Grade 1	23	14% (9, 21)	9	10% (5, 19)	3	10% (2, 27)	11	24% (13, 39)
Grade 2	18	11% (7, 17)	10	12% (6, 20)	3	10% (2, 27)	5	11% (4, 24)
Grade 3	121	75% (67, 81)	68	78% (68, 86)	23	79% (60, 92)	30	65% (50, 79)
Data not available	18		11		3		4	
Invasive cancer	1045		469		234		342	
Tumor diameter (mm)								
Mean ± SD	15 mm ± 10		15 mm ± 9		16 mm ± 10		15 mm ± 10	
Median (IQR)	13 mm (9, 19)		12 mm (9, 18)		13 mm (10, 20)		13 mm (9, 19)	
Data not available	23		12		5		6	
Histological grade								
Grade 1	288	28% (25, 30)	103	23% (19, 27)	73	32% (26, 38)	112	33% (28, 38)
Grade 2	519	50% (47, 54)	216	47% (43, 52)	124	54% (47, 61)	179	52% (47, 58)
Grade 3	222	22% (19, 24)	138	30% (26, 35)	33	14% (10, 20)*	51	15% (11, 19)*
Data not available	16		12		4			
Lymph node status								
Negative	797	80% (77, 82)	353	78% (74, 82)	182	81% (76, 86)	262	81% (76, 85)
Data not available	47		19		10		18	
Hormonal status								
ER positive	919	91% (89, 93)	387	86% (82, 89)	215	95% (92, 98)*	317	95% (92, 97)*
Data not available	35		18		8		9	
PR positive	730	73% (70, 75)	303	67% (62, 71)	173	78% (72, 83)	254	77% (72, 81)
Data not available	39		16		11		12	

Unless otherwise specified, data are the number of patients and 95% confidence intervals in parentheses

*DCIS* ductal carcinoma in situ, *NST* no special type, *ILC* invasive lobular carcinoma, *ER* estrogen receptor, *PR* progesterone receptor, *SD* standard deviation, *IQR* interquartile range

*p < 0.001, compared with true

Among invasive cancers, 30% (138/457) of true cancers were histological grade 3, compared with 14% (33/230) of missed and 15% (51/342) of minimal signs (*p* < 0.001 for missed or minimal signs versus true). We observed no differences in mean histopathologic tumor diameter or lymph node status between the groups. The percentage of ER+ cases was 86% (387/451) for true and 95% for both missed (215/226) and minimal signs (317/333) (*p* < 0.001 for missed or minimal signs versus true). Sixty-seven percent (303/453) of true, 78% (173/223) of missed, and 77% (254/330) of minimal sign cancers were PR+ (*p* = 0.004 for missed versus true and *p* = 0.002 for minimal signs versus true) (Table 2).

We observed the highest percentage of DCIS, 60% (145/242) among cases presenting as calcifications (Table 3). Masses, asymmetries, and distortions were mainly invasive carcinoma of NST, ranging from 71% (69/97) for distortions to 83% (646/777) for masses. We observed 21% (20/97) for ILC among distortions, compared with 7% (54/777) for masses and 2% (5/242) for calcifications. Among invasive cancers, distortions were associated with the lowest percentage of histological grade 3 tumors (8%, 7/92) and calcifications were associated with the highest (41%, 39/94). The mean mammographic diameter for invasive cancers ranged from 15 mm ± 8 (masses) to 36 mm ± 26 (calcifications). Mean histopathologic tumor diameter ranged from 12 mm ± 11 (calcifications) to 20 mm ± 15 (distortions).

**Table 3:** Histopathological tumor characteristics stratified by mammographic findings

	Mass		Calcifications		Asymmetry		Distortion	
	*n* = 777		*n* = 242		*n* = 106		*n* = 97	
Histopathological type								
DCIS	23	3% (2, 4)*	145	60% (53, 66)	9	9% (4, 16)*	3	3% (1, 9)*
Invasive carcinoma of NST	646	83% (80, 86)*	85	35% (29, 42)	78	74% (64, 82)*	69	71% (61, 80)*
ILC	54	7% (5, 9)**	5	2% (1, 5)	14	13% (7, 21)*	20	21% (13, 30)*
Other invasive carcinomas	54	7% (5, 9)	7	3% (1, 6)	5	5% (2, 11)	5	5% (2, 12)
Invasive cancer	754		97		97		94	
Histological grade								
Grade 1	211	28% (25, 32)	13	14% (8, 23)	26	28% (1, 38)	38	41% (31, 52)*
Grade 2	378	51% (47, 54)	42	45% (34, 55)	51	54% (44, 65)	47	51% (40, 62)
Grade 3	158	21% (18, 24)*	39	41% (31, 52)	17	18% (11, 27)*	7	8% (3, 15)*
Data not available	7		3		3		2	
Lymph node status								
Negative	583	80% (77, 83)	73	85% (76, 92)	74	80% (70, 87)	67	74% (64, 83)
Data not available	28	28	11		4		4	
Hormonal status								
ER positive	661	90% (88, 92)	75	85% (76, 92)	89	95% (88, 98)	92	100% (96, 100)*
Data not available	21		9		3		2	
PR positive	532	73% (70, 76)*	47	54% (43, 65)	69	73% (63, 82)	80	87% (78, 93)*
Data not available	24		10		3		2	
Mammographic diameter (mm)								
Mean ± SD		15 mm ± 8*^,^ **		36 mm ± 26		17 mm ± 16*		22 mm ± 15*
Median (IQR)		13 mm (9, 18)		30 mm (17, 50)		13 mm (9, 20)		20 mm (14, 25)
Data not available	45		7		7		4	
Tumor diameter (mm)								
Mean ± SD		15 mm ± 8		12 mm ± 11		16 mm ± 12		20 mm ± 15*
Median (IQR)		12 mm (9, 18)		9 mm (5, 17)		14 mm (8, 17)		15 mm (12, 22)
Data not available	11		9		0		2	

Unless otherwise specified, data are the number of patients and 95% confidence interval in parentheses

*DCIS* ductal carcinoma in situ, *NST* no special type, *ILC* invasive lobular carcinoma, *ER* estrogen receptor, *PR* progesterone receptor, *SD* standard deviation, *IQR* interquartile range

*p < 0.001, compared with calcifications; **p < 0.001, compared with distortion

For masses, the percentage of histological grade 3 invasive cancer was higher for true than for missed and minimal sign screen-detected breast cancer (*p* < 0.001 for true compared with both missed and minimal signs); otherwise, we observed no differences in histopathological tumor characteristics stratified by review classification categories and mammographic findings (Table 4). We observed no differences for mammographic findings, histologic tumor type, diameter, and grade for minimal signs, actionable versus non-actionable tumors (Table 5 in the Appendix).

**Table 4:** Histopathological type, tumor diameter, and histological grade for mammographic findings stratified by review categories

	True		Missed		Minimal signs	
Mass	*n* = 336		*n* = 185		*n* = 256	
Histopathological type						
DCIS	9	3% (1, 5)	7	4% (2, 8)	7	3% (1, 6)
Invasive carcinoma of NST	291	87% (83, 90)	149	81% (74, 86)	206	81% (75, 85)
ILC	17	5% (3, 8)	11	6% (3, 10)	26	10% (7, 15)
Other invasive carcinomas	19	6% (3, 9)	18	10% (6, 15)	17	7% (4, 10)
Tumor diameter of invasive cancer						
Mean ± SD	14 mm ± 8		15 mm ± 9		14 mm ± 8	
Median (IQR)	12 mm (9, 18)		13 mm (10, 18)		12 mm (9, 19)	
Not available	5		3		3	
Histological grade of invasive cancer						
Grade 1	72	22% (18, 27)	61	35% (28, 42)	78	31% (26, 38)
Grade 2	150	47% (41, 52)	96	54% (47, 62)	132	53% (47, 59)
Grade 3	99	31% (26, 36)	20	11% (7, 17)*	39	16% (11, 21)*
Not available	6		1		0	
Asymmetry	*n* = 58		*n* = 15		*n* = 33	
Histopathological type						
DCIS	5	9% (3, 19)			4	12% (3, 28)
Invasive carcinoma of NST	46	79% (67, 89)	10	67% (38, 88)	22	67% (48, 82)
ILC	4	7% (2, 17)	15	33% (12, 62)	5	15% (5, 32)
Other invasive carcinomas	3	5% (1, 14)			2	6% (1, 20)
Tumor diameter (mm) of invasive cancer						
Mean ± SD	14 mm ± 11		20 mm ± 9		16 mm ± 14	
Median (IQR)	13 mm (8, 16)		19 mm (15, 24)		14 mm (8, 16)	
Not available						
Histological grade of invasive cancer						
Grade 1	10	20% (10, 34)	2	13% (2, 41)	14	48% (29, 68)
Grade 2	26	52% (37, 66)	10	67% (38, 88)	15	52% (33, 71)
Grade 3	14	28% (16, 43)	3	20% (4, 48)		
Not available	3					
Calcifications	*n* = 128		*n* = 46		*n* = 68	
Histopathological type						
DCIS	83	65% (56, 73)	23	50% (35, 65)	39	57% (45, 69)
Invasive carcinoma of NST	39	31% (23, 39)	21	46% (31, 61)	25	37% (25, 49)
ILC	3	2% (1, 7)	1	2% (0, 12)	1	2% (0, 8)
Other invasive carcinomas	3	2% (1, 7)	1	2% (0, 12)	3	4% (1, 12)
Tumor diameter of invasive cancer						
Mean ± SD	13 mm ± 12		11 mm ± 10		12 mm ± 9	
Median (IQR)	10 mm (6, 15)		6 mm (4, 11)		10 mm (5, 19)	
Not available	5		2		2	
Histological grade of invasive cancer						
Grade 1	6	14% (5, 27)	3	14% (3, 36)	4	14% (4, 32)
Grade 2	18	41% (26, 57)	10	48% (26, 70)	14	48% (29, 68)
Grade 3	20	45% (30, 61)	8	38% (18, 62)	11	38% (21, 58)
Not available	1		2			
Distortion	*n* = 43		*n* = 20		*n* = 34	
Histopathological type						
DCIS	1	2% (0, 12)	2	10% (1, 32)		
Invasive carcinoma of NST	29	67% (52, 81)	14	70% (46, 88)	26	77% (59, 89)
ILC	11	26% (14, 41)	4	20% (6, 44)	5	15% (5, 31)
Other invasive carcinomas	2	5% (1, 16)			3	9% (2, 24)
Tumor diameter (mm) of invasive cancer						
Mean ± SD	19 mm ± 13		21 mm ±14		20 mm ± 17	
Median (IQR)	16 mm (10, 22)		17 mm (12, 22)		15 mm (12, 22)	
Not available	1				1	
Histological grade of invasive cancer						
Grade 1	15	37% (2, 53)	7	41% (18, 67)	16	47% (30, 65)
Grade 2	22	54% (37, 69)	8	47% (23, 72)	17	50% (32, 68)
Grade 3	4	10% (3, 23)	2	12% (2, 36)	1	3% (0, 15)
Not available	1		1			

Unless otherwise specified, data are the number of patients and 95% confidence intervals in parentheses

*DCIS* ductal carcinoma in situ, *NST* no special type, *ILC* invasive lobular carcinoma, *ER* estrogen receptor, *PR* progesterone receptor, *SD* standard deviation, *IQR* interquartile range

*p < 0.001, compared with true.

## Discussion

In this informed, consensus-based review of mammograms from prior screening and diagnosis of 1225 women with screen-detected cancer, radiologists classified 46% as true, 22% as missed, and 32% as minimal signs. The most frequent mammographic finding at diagnosis was a mass for all classification categories; no statistically significant differences were observed between the classification categories regarding mammographic findings at diagnosis. At prior screening, the most frequent mammographic finding for missed cancer was a mass, whereas for minimal sign cancer, it was asymmetry. The majority of asymmetries at prior screening progressed into masses by the time of diagnosis. Mammographic density did not differ between the review classification categories. True invasive cancers were more often histological grade 3 and had less favorable hormonal status than missed and minimal sign invasive cancers.

Our findings support results from other retrospective, informed review studies of screen-detected breast cancer. In a study by Ikeda et al [8], findings at the later cancer site, obvious or non-specific, were observed retrospectively in 67% of screen-detected breast cancers. Van Breest Smallenburg et al [9] found 21% of screen-detected breast cancer to be missed and 22% with non-specific minimal signs at informed review, and Broeders et al [7] identified findings on prior screening mammograms in 53% of screen-detected cancers. However, all three studies included screen-film mammography (SFM). In an informed review from BreastScreen Norway, 12% of screen-detected breast cancers were classified as  missed and 9% minimal sign actionable for screening with SFM [6] and 10% missed and 9% minimal sign actionable for screening with DM [1]. However, the review procedures and definition of classification groups differed between the studies, making comparison challenging. In experimental review studies of interval breast cancer exploring different study designs, the percentage of cancers classified as missed differed largely depending on review procedure and number of radiologists. Hofvind et al [3] found the percentage of missed interval breast cancer ranging from 1% (mixed, blinded individual review) to 34% (informed, consensus-based) and 36% (informed individual). In a study by Ciatto et al [4], the proportion of missed (screening error) cancers varied from 24% in a simulated blinded review to 42% in a simulated fully informed review. Further, studies have demonstrated that the proportion of missed cancer is affected by how close the study setting is to a normal screening setting [25, 26]. Easy understandable standardized definitions and recommendations on classification groups and review procedure are needed to enable future comparison of results from reviews.

True cancers include tumors detected at an early stage, reflected by the higher percentage of DCIS among true versus missed cancers. However, true cancers may also be fast-growing tumors, with less favorable tumor characteristics [27, 28]. This is illustrated in our study by the larger percentage of histological grade 3 invasive cancer among true compared with missed and minimal sign cancers. The largest discrepancy in the diameter of mammographic findings versus histopathological tumor diameter was observed among calcifications (36 mm ± 26 versus 12 mm ± 11). Invasive cancers presenting as calcifications were also smaller and had a higher histological grade than cancers presenting with other mammographic findings. This may reflect small, aggressive invasive tumors imbedded in a larger calcified area, possibly of DCIS. However, we cannot elaborate on this hypothesis because only the diameter of the invasive component is coded in the database at the CRN. The larger tumor diameter and higher percentage of tumors with low/intermediate histological grade among distortions may be related to the increased frequency of ILC among distortions. ILC is generally of low/intermediate histological grade and larger at diagnosis than other invasive carcinomas [29–31].

A mass was the most frequent finding on prior mammograms of missed cancers, and a special emphasis on masses at screening might be reasonable. Masses might be misinterpreted as benign, in particular if retrospectively visible on more than one prior screening exam or if not having spiculated margins [18, 32]. Further, the mean diameter of mammographic findings of missed cancer was 11 mm, which usually is regarded to be above the limit for visual perception. This could indicate that a certain proportion of cancers was missed due to misinterpretation at screen reading or dismissed at consensus. The high frequency of asymmetries on priors of missed and minimal signs developing into masses at the screening exam leading to diagnosis of cancer is in line with other studies; increased awareness of asymmetries may be useful to reduce the burden of missed cancers at screening [32, 33]. However, asymmetries are common and most often represent glandular tissue, in particular if visible only in one view. Thus, radiologists should be attentive and should avoid an unreasonably increase in the recall rate for such findings. Evaluating more than one prior screening exam may be valuable in this respect. Moreover, a recent study showed that increasing the recall rate mainly increased detection of low-grade and not high-grade cancer [34]. This is consistent with our results demonstrating a higher proportion of tumors of low and intermediate histological grade among missed/minimal signs than true; an increased recall rate would probably reduce the proportion of missed and minimal signs.

Our study has some limitations. First, the review was consensus-based and all images were available—this design yields the highest percentage of missed cancers in review studies [3–5]. This limits the external generalizability of our results. Second, our study was performed at 16 breast centers with images from a span of approximately 10 years, and major heterogeneity in the combinations of PACS, workstations, and mammographic equipment. As a result, the image quality and presentation differed between centers, which might have influenced the assessment of review classifications, mammographic findings, and density. Third, the consensus panel included five radiologists: one who participated in all the reviews and four who only participated in one session at their own and one at the paired center. Although we presented and communicated the general instructions and classification systems to all radiologists at the start of each review, some differences in interpretation and assessment between radiologists are likely to have occurred. However, we included mammograms and radiologists from all breast centers in Norway, and our study is as far as we are aware of, the largest reported in peer-reviewed journals, which we consider as strength. Finally, we assessed mammographic density from the screening mammograms at diagnosis. This might have biased the association between review categories (classified from prior screening mammograms 2 years earlier) and mammographic density, as the women’s breast density might have decreased during the 2 years.

The study confirmed our hypothesis that mammographic findings and histopathologic characteristics differed between true and missed screen-detected breast cancers in BreastScreen Norway. However, we did not identify differences in mammographic density between review classification categories. One main goal of the classification of missed cancers was quality improvement for radiologists’ performance and the program. We would like to emphasize that the review and study setting differed substantially from real-life screening settings. Visible findings on priors were not necessarily indicative of a screening failure. Recalling all women with subtle findings would increase the rate of false positive recalls and probably also the detection of small, low proliferation tumors (overdiagnosis). This is important to keep in mind during audits and when exploring medicolegal aspects of mammographic screening.

## Appendix

**Table 5:** Mammographic findings and histopathological characteristics for minimal signs actionable versus minimal signs non-actionable

	Minimal signs actionable		Minimal signs non-actionable	
	*n* = 192		*n* = 200	
Diagnostic mammograms				
Mass	140	73% (66, 79)*	116	58% (51, 65)
Calcifications	24	13% (8, 18)	44	22% (17, 28)
Asymmetry	13	7% (4, 11)	20	10% (6, 15)
Distortion	15	8% (4, 13)	19	10% (6, 14)
Associated features			1	0.5% (0, 3)
Mammographic diameter				
Mean ± SD		20 mm ± 17		18 mm ± 15
Median (IQR)		15 mm (10, 22)		14 mm (9, 20)
Data not available	14		16	
Prior mammograms				
Mass	13	7% (4, 11)	6	3% (1, 6)
Calcifications	32	17% (12, 23)	48	24% (18, 31)
Asymmetry	117	61% (54, 68)	119	60% (52, 66)
Distortion	29	15% (10 21)	26	13% (9, 19)
Associated features	1	0.5% (0, 3)	1	0.5% (0, 3)
Histopathological type				
DCIS	20	10% (7, 16)	30	15% (10, 21)
Invasive carcinoma of NST	138	72% (65, 78)	141	71% (64, 77)
ILC	19	10% (6, 15)	19	10% (6, 14)
Other	15	8% (4, 13)	10	5% (2, 9)
Invasive cancer	n = 172		n = 170	
Tumor diameter				
Mean ± SD		16 mm ± 11		14 mm ± 9
Median (IQR)		13 mm (9, 19)		13 mm (8, 20)
Data not available	4		2	
Histological grade				
Grade 1	54	31% (25, 39)	58	34% (27, 42)
Grade 2	96	56% (48, 63)	83	49% (41, 57)
Grade 3	22	13% (8, 19)	29	17% (12, 24)

Unless otherwise specified, data are the numbers of patient and 95% confidence intervals in parentheses

*DCIS* ductal carcinoma in situ, *NST* no special type, *ILC* invasive lobular carcinoma, *SD* standard deviation, *IQR* interquartile range

**p* = 0.002, compared with minimal sign actionable

## Abbreviations

BI-RADS: Breast Imaging-Reporting and Data System
CI: Confidence interval
CRN: Cancer Registry of Norway
DCIS: Ductal carcinoma in situ
DM: Digital mammography
ER: Estrogen receptor
HER-2: Human epidermal growth factor receptor 2
ILC: Invasive lobular carcinoma
IQR: Interquartile range
NST: No special type
PACS: Picture archiving and communication system
PR: Progesterone receptor
SD: Standard deviation
